# Effects of Drip Irrigation-Fertilization on Growth, Flowering, Photosynthesis and Nutrient Absorption of Containerized Seedlings of *Hibiscus syriacus* L. (Haeoreum)

**DOI:** 10.3390/plants12122293

**Published:** 2023-06-12

**Authors:** Eon-Ju Jin, Jun-Hyuck Yoon, Hyeok Lee, Hae-Yun Kwon, Han-Na Shin, Seong-Hyeon Yong, Myung-Suk Choi

**Affiliations:** 1 Forest Biomaterials Research Center, National Institute of Forest Science, Jinju 52817, Republic of Korea; jinej85@korea.kr (E.-J.J.); jhyoon@korea.kr (J.-H.Y.); hyeok0902@korea.kr (H.L.); 2 Forest Medicinal Resources Research Center, National Institute of Forest Science, Yeongju 36040, Republic of Korea; kwonhy05@korea.kr; 3 Division of Special Forest Resources, Department of Forest Bio-Resources, National Institute of Forest Science, Suwon 16631, Republic of Korea; hanna193@korea.kr; 4 Division of Forest Environmental Resources and Institute of Agriculture and Life Science, Gyeongsang National University, Jinju 52828, Republic of Korea; ysh1820@naver.com

**Keywords:** container nursery, chlorophyll fluorescence, ornamental tree, photosynthetic ability, quality index, nutrient vector analysis

## Abstract

The amount of irrigation and fertilization should be considered first for the production and standardization of high-quality *H. syriacus* L. seedlings using container seedlings. This study was conducted to investigate the optimal conditions suitable for container cultivation of hibiscus by analyzing growth and physiological responses according to the control of irrigation and fertilization. Therefore, in this study, *H. syriacus* L. for. *Haeoreum* (3-year-old hardwood cutting propagation), a fast-growing, was transplanted into a 40 L container. The irrigation amount per container was adjusted (0.2, 0.3 and 0.4 ton/yr/tree), and the amount of fertilizer applied (0, 69.0, 138.0 and 207.0 g/yr/tree). The growth rate according to the irrigation-fertilization treatment was higher in the 0.3 ton-138.0 g/yr/tree irrigation-fertilization treatment (*p* < 0.001). Total biomass yield and seedling quality index (SQI) were highest in the 0.3 ton-138.0 g/yr/tree irrigation-fertilization treatment (*p* < 0.001). The higher the fertilization concentration, the faster the flowering and the longer the flowering. The photosynthetic capacity of *H. syriacus* L. was reduced in bare root seedling cultivation and container-non-fertilized treatment. The chlorophyll fluorescence response was also affected by bare root cultivation and containerized seedling cultivation fertilization. Nutrient vector diagnosis showed “nutritional suitability” in the 0.3 ton-138.0 g/yr/tree treatment. Overall, containerized seedling cultivation was superior in growth, photosynthetic performance, photochemical efficiency, and nutrient storage capacity compared to bare root cultivation. These results be expected to contribute not only to the industrial production of excellent container seedlings of *H. syriacus* L. but also to the production of other woody plants.

## 1. Introduction

*Hibiscus syriacus* L. has grown numerous varieties through hybridization over a long period, and about 200 species are distributed worldwide [1]. It is an ornamental tree with high research value as firewood that smokes [2]. *H. syriacus* L. research began in the early 1970s. From the late 1980s, the Korean *H. syriacus* L. research society introduced breeding, selective breeding, crossbreeding between species, breeding mutations and polyploidy by treatment with radioactive isotopes and colchicine, and varieties were developed using tissue culture, etc. Since 2008, the Korea Forest Service has continuously promoted the ‘Citizen-friendly *H. syriacus* Expansion Basic Plan’ and created *H. syriacus* L. flower gardens and roads throughout the country. In addition, it can be seen that the leaves do not fall until late in early winter, and the leaves are green. Recently, efforts have been made to increase various landscaping uses as indoor plants grown in pots [3].

Standardization of container seedling cultivation and mechanization of working processes are well established in Europe, the United States and Canada, producing high-quality nurseries. Through this, industrialization in landscape tree production has been realized [4]. Container-grown trees can be planted without root damage, so they are easy to establish after transplantation, are less subject to seasonal restrictions on planting time, and standardized production is possible compared to bare root seedling cultivation, maximizing the added value of landscape trees. However, most of *H. syriacus* production in Korea is currently bare root seedling cultivation. In this bare root seedling cultivation method, poor drainage, damage from frost damage, and insufficient maintenance were causes that increased the defect rate in landscaping construction [5]. 

*H. syriacus* L. studies that have been conducted so far have mainly selected native species focused on flower color or studies related to cultivating various varieties for differentiation [6,7,8,9], studies on suitable production and management systems for containerized seedling cultivation for urban greening are insufficient.

In containerized seedling cultivation, the first thing to be considered is irrigation-fertilization management, which are essential elements for tree growth [10]. In particular, since containerized seedling cultivation grows trees within a limited container volume, growth environment control such as light, moisture, temperature, type of container, bed soil, and fertilization technology have significant effects [10,11,12]. Water is a significant component that accounts for about 80% of the plant’s weight. It promotes biochemical reactions and the growth of various metabolic processes in plants, including photosynthesis, and also affects cell expansion [13]. Stress due to excess/lack of water leads to the production of poor trees, and leaf and stem growth are affected due to water stress. Leaf area decreases, reducing transpiration and photosynthetic capacity [14,15]. In general, containerized landscape trees are ideally maintained in the soil within the containers so that all irrigation is applied to the trees. Containerized seedling cultivation for most small trees is irrigated with an overhead sprinkler method. Only 20–40% of the total irrigation amount is retained in the container [16]. In the case of containerized seedling cultivation for medium and large trees, a micro irrigation system using a dropper is generally used to minimize water waste [17]. Optimal irrigation levels and methods for containerized tree cultivation should be determined by optimizing irrigation uniformity and fully considering the moisture requirements of containerized trees [18]. In this regard, studies have been conducted on moisture control suitable for the specifications of each tree species for seedlings. However, it is not easy to investigate the quantification of irrigation amount by growth stage considering the physiology of trees [19]. In general, containerized landscape trees are ideally maintained in the soil within the containers so that all irrigation is applied to the trees. Also, in containerized seedling cultivation, fertilization is performed in parallel with irrigation [20]. 

Nitrogen and potassium are required for the growth of various types of nutrients, which are essential elements for the growth of trees. Eventually, this leads to decreased growth [21]. Scagel et al. [22] reported that plant growth and flowering are affected by the N content of plants. A high N fertilization rate increased the N content in the plant and improved the flowering quantity and flower size. However, environmental pollution problems due to excessive fertilization continue to occur in arable land [23]. Irrigation (period, frequency and time) and fertilization are generally driven primarily by the grower’s experience, even when landscape trees are grown in containers [24]. Due to this, it is impossible to measure the amount of water required by trees quantitatively, so trial and error are experienced in adjusting the amount of irrigation. Appropriate fertilization without damage to growth is also required, rather than a uniform fertilization technique.

Seedling production of *H. syriacus* L. is mainly dependent on bare root cultivation, or fertilization is important because it is a fertile landscaping tree, and the importance of fertilization is low due to its strong drought tolerance. In addition, fertilization and irrigation are considered separate container seedling cultivation factors when producing *H. syriacus* L. seedlings. Therefore, in this study, the following research hypotheses are set. First, irrigation and fertilization complex factors do not affect most growth-related factors, such as growth and biomass. Second, irrigation and fertilization complex factors will not significantly affect photosynthetic capacity and chlorophyll fluorescence response. Finally, container seedling cultivation by optimization of irrigation and fertilization factors will not be significantly more efficient than open field cultivation.

Therefore, this study was conducted to identify the optimal production conditions for high-quality *H. syriacus* L. container seedlings. (1) In container seedling cultivation, the growth rate and quality of seedlings were compared and analyzed according to the amount of irrigation-fertilization concentration. (2) The flowering characteristics according to the amount of irrigation-fertilization concentration were investigated, (3) the effect of the amount of irrigation-fertilization treatment on the photosynthesis parameters and chlorophyll fluorescence response was analyzed, Finally, (4) the appropriate amount of fertilizer suitable for the production of healthy container seedlings was determined through nutrient vector analysis. 

## 2. Results

### 2.1. Growth Characteristics According to Fertilization and Irrigation

*H. syriacus* L. growth was significantly affected by fertilization concentration (*p* < 0.05) rather than irrigation (*p* ≥ 0.05), showing differences between treatments (Table 1). Compared to rare root seedling cultivation, the height and root collar diameter growth increased in containerized seedling cultivation. The growing root collar diameter affected the interaction between irrigation-fertilization (*p* < 0.05). Regarding height and root collar diameter, the 0.3 ton-138.0 g/yr/tree reatment showed a significant (*p* < 0.01) high growth rate, and the growth rate decreased in the 207.0 g/yr/tree treatments with the highest fertilization concentration. The H/D ratio showed the lowest value in the 138.0 g/yr/tree (4.23~4.75 cm·mm^−1^) treatment. Also, it was 15.3% lower than bare root seedling cultivation (5.53 ± 0.69 cm·mm^−1^) and 19.8% lower than container-non-fertilized treatment (5.79~5.92 cm·mm^−1^).

### 2.2. Biomass Amount, T/R Rate and Quality Index According to Fertilization and Irrigation

**Figure 1:**
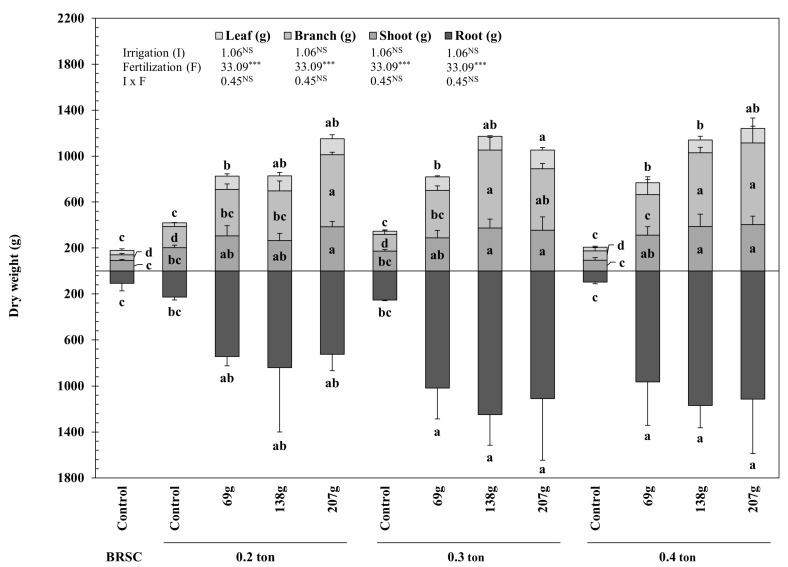
Biomass production of measured from 2020 to 2021 on containerized seedlings of *H. syriacus* L. grown under different three irrigation and four fertilization treatments. BRSC is an abbreviation for bare root seedling cultivation. ^NS^ *p* ≥ 0.05, *** *p* < 0.001. Values with different letters in a column indicate statistical differences three irrigation and four fertilization treatments at the 5% levels by Duncan’s multiple range test.

The amount of biomass of *H. syriacus* L. also showed a significant (*p* < 0.001) effect by fertilization rather than irrigation (*p* ≥ 0.05) and showed a higher value in the container-grown fertilization treatment than in the bare root seedling cultivation (Figure 1). The total biomass amount of *H. syriacus* L. was significantly (*p* < 0.001) high at 0.3 ton-138.0 g/yr/tree (2422.2 g), showing a value that increased by 88.2% compared to bare root seedling cultivation. In addition, it showed a higher yield of 78.7% than container-non-fertilized treatment (304.6~647.1 g). In the case of 0.3 ton-138.0 g/yr/tree of biomass by part, the production of biomass in the underground part compared to the above-ground part increased by 6.1%, whereas in all other treatments, the amount of biomass in the above-ground part compared to the underground part was significantly higher (*p* < 0.01). In particular, in the container-non-fertilized treatment, the amount of biomass in the above-ground part increased by 40.3% compared to the underground part, showing overgrowth in the above-ground part.

**Figure 2:**
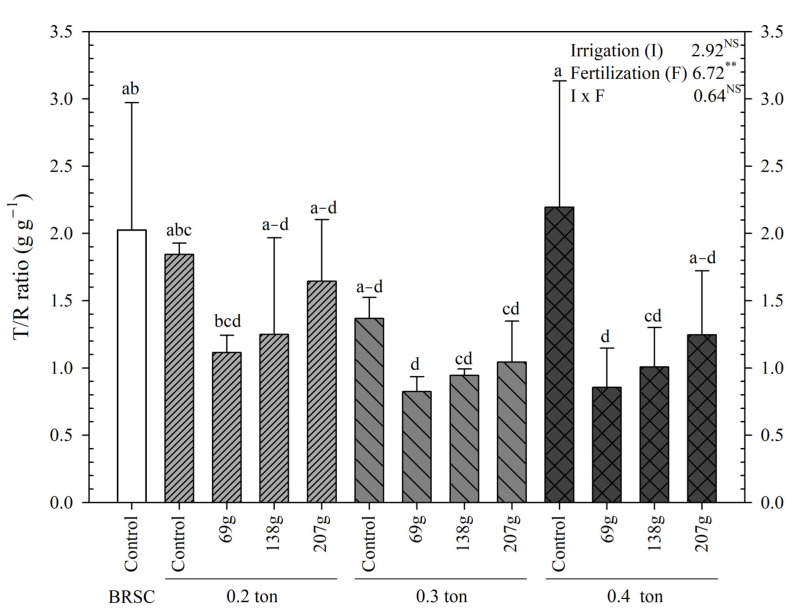
T/R ratio of measured from 2020 to 2021 on containerized seedlings of *H. syriacus* L. grown under different three irrigation and four fertilization treatments. BRSC is an abbreviation for bare root seedling cultivation. ^NS^ *p* ≥ 0.05, ** *p* < 0.01. Values with different letters in a column indicate statistical differences three irrigation and four fertilization treatments at the 5% levels by Duncan’s multiple range test.

**Figure 3:**
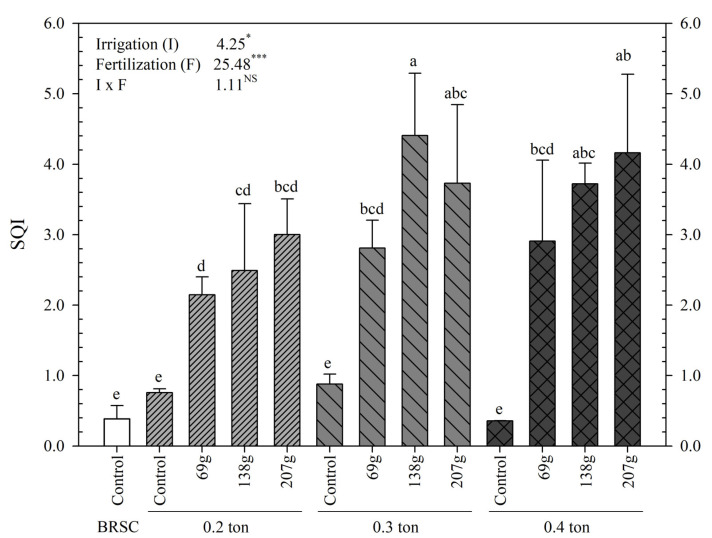
SQI ratio of measured from 2020 to 2021 on containerized seedlings of *H. syriacus* L. grown under three different irrigation and four fertilization treatments. BRSC is an abbreviation for bare root seedling cultivation. BRSC is an abbreviation for bare root seedling cultivation. ^NS^ *p* ≥ 0.05, * *p* < 0.05, *** *p* < 0.001. Values with different letters in a column indicate statistical differences three irrigation and four fertilization treatments at the 5% levels by Duncan’s multiple range test.

The T/R ratio was high in bare root seedling cultivation (2.03) and container-non-fertilized treatment (1.37~2.20) and tended to increase as fertilization increased. The treatments with the lowest T/R rates were the 0.3 ton-69.0 g/yr and 138.0 g/yr/tree treatments, 59.6% and 53.7% lower than the bare root seedling cultivation and 54.4% and 48.7% lower than the container-non-fertilized treatment, respectively (Figure 2). SQI showed a significantly (*p* < 0.001) high value at 0.3 ton-138.0 g/yr/tree (4.40) and was 91.1% and 84.8% higher than bare root seedling cultivation (0.39) and container-non-fertilized treatment (0.36–0.88), respectively (Figure 3).

### 2.3. Flowering Characteristics According to Fertilization and Irrigation

**Figure 4:**
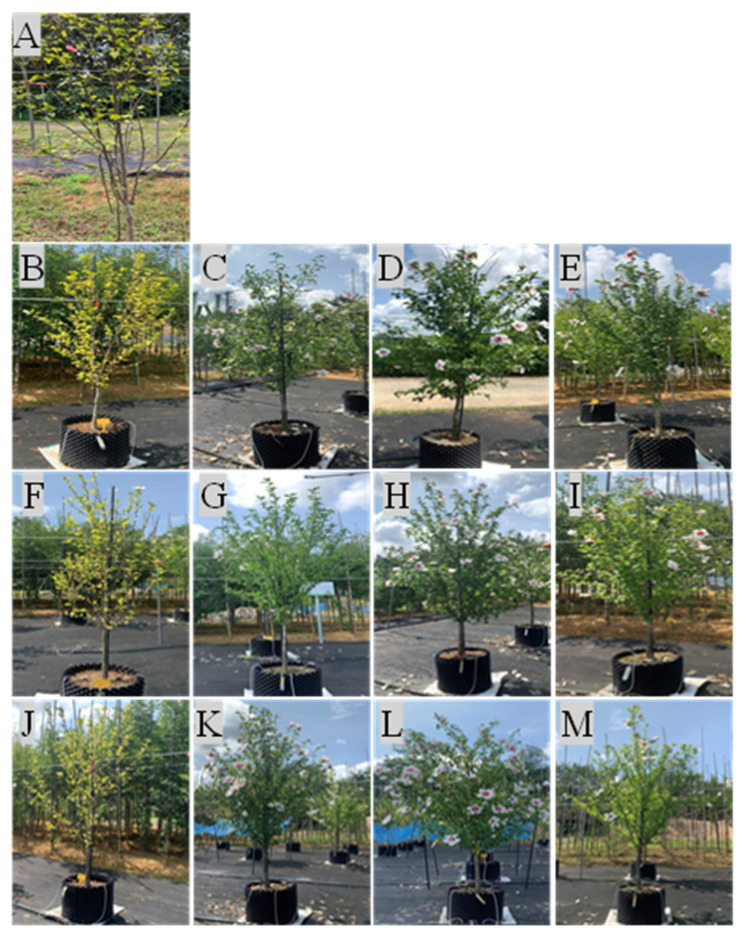
Visual appearance of measured from 2020 to 2021 on containerized seedlings of *H. syriacus* L. grown under three irrigation and four fertilization treatments. (**A**): Control in BRSC, (**B**–**E**): 0.2 ton-(Control, 69.0, 138.0, 207.0 g), (**F**–**I**): 0.3 ton-(Control, 69.0, 138.0, 207.0 g), (**J**–**M**): 0.4 ton-(Control, 69.0, 138.0, 207.0 g). BRSC is an abbreviation for bare root seedling cultivation.

Irrigation-fertilization were shown to have significant effects on *H. syriacus* L. flowering (Table 2, Figure 4). The time required for flowering was faster as the amount of application increased, with 78.4 ± 0.3 days at 0.2 ton-207.0 g/yr/tree treatment, flowering the earliest, followed by 78.7 ± 0.2 days at 0.3 ton-138.0 g/yr/tree treatment, faster than other treatments. On the other hand, the flowering time of bare root seedling cultivation was 97.8 ± 0.7 days, 19.4 days later than 0.2 ton-207.0 g/yr/tree. The average number of days required for flowering in the container-non-fertilized treatment was 108.3 days, 10.5 days later than bare root seedling cultivation and 29.9 days later than 0.2 ton-207.0 g/yr/tree treatment. 

Flowering speed also increased as the amount of fertilization increased, and there was a significant effect between irrigation (*p* < 0.05) and fertilization (*p* < 0.001), but there was no interaction. The flowering rate was the fastest at 0.3 ton-207.0 g/yr/tree (4.1 ± 0.5) treatment, followed by 0.3 ton-138.0 g/yr/tree (3.5 ± 0.5) treatment. The total flowering amount was the highest at 0.3 ton-207.0 g/yr/tree (468.7 ± 57.5 ea) treatment, followed by 0.3 ton-138.0 g/yr/tree (443.3 ± 70.7 ea) treatment. As for the flowering period, the interaction between irrigation-fertilization was found to be significant (*p* < 0.001), 0.3 ton-207.0 g/yr (91.8 ± 0.3 days) treatment, 0.3 ton-138.0 g/yr (88.4 ± 1.3 days) treatment continued flowering for 20.2~23.8 days compared to bare root cultivation and 40.3~43.7 days compared to container-non-fertilized treatment.

### 2.4. Changes in Photosynthesis Parameters According to Fertilization and Irrigation

**Figure 5:**
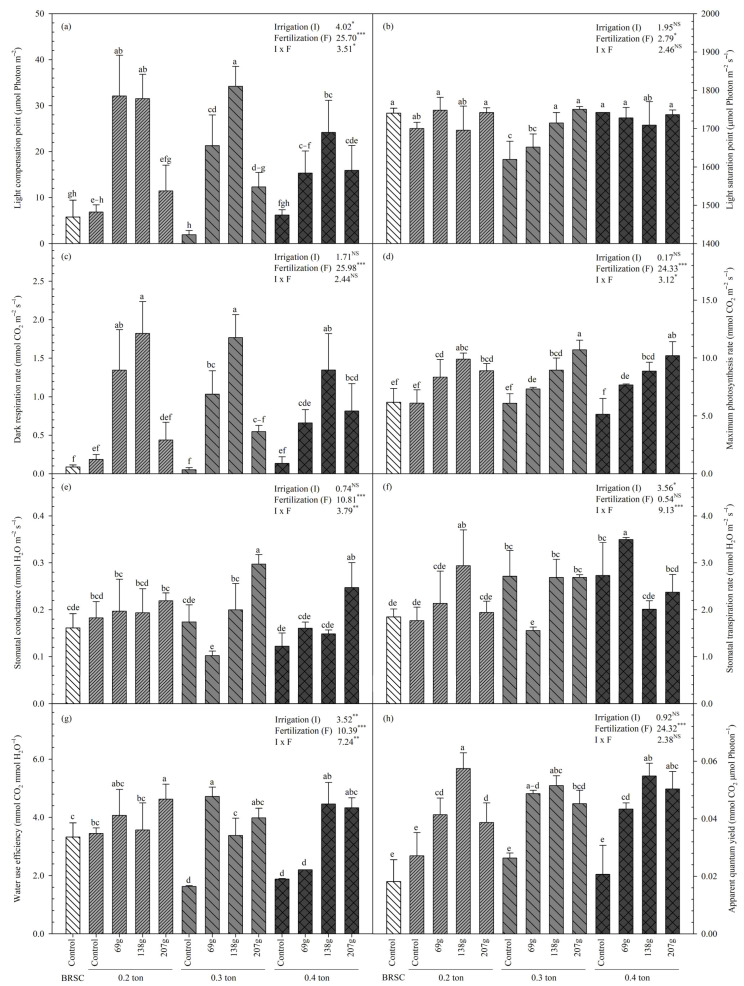
Photosynthetic parameters (**a**–**h**) from 2020 to 2021 on containerized seedlings of *H. syriacus* L. grown under different three irrigation and four fertilization treatments. BRSC is an abbreviation for bare root seedling cultivation. ^NS^ *p* ≥ 0.05, * *p* < 0.05, ** *p* < 0.01, *** *p* < 0.001. Values with different letters in a column indicate statistical differences three irrigation and four fertilization treatments at the 5% levels by Duncan’s multiple range test.

The light compensation points also showed a significant interaction (*p* < 0.05) effect according to irrigation (*p* < 0.05) and fertilization (*p* < 0.001) (Figure 5a). The container-non-fertilized treatment (1.94~6.86 mmol·m^−2^·s^−1^) had the lowest light compensation point, which was 22.1% lower than that of bare seedling cultivations. The treatment with the highest light compensation point was 0.3 ton-138.0 g/yr/tree (34.2 ± 4.34 mmol·m^−2^·s^−1^), 86.8% higher than container-non-fertilized treatment.

The light saturation point was also the lowest in container-non-fertilized treatment, and it tended to increase as fertilization increased (Figure 5b). 

The dark respiration rate showed a significant difference (*p* < 0.001) according to fertilization treatment, 0.2 ton-138.0 g/yr/tree (1.82 ± 0.41 mmol·m^−2^·s^−1^) and 0.3 ton-138.0 g/yr/tree (1.77 ± 0.30 mmol·m^−2^·s^−1^) treatments showed a higher value than other treatments (Figure 5c). 

The maximum photosynthetic rate increased significantly (*p* < 0.001) as the amount of fertilization increased, and a significant interaction (*p* < 0.05) between irrigation-fertilization was also shown. In the case of 0.3 ton-207.0 g/yr/tree (10.20 ± 1.20 mmol CO_2_·m^−2^·s^−1^) treatment, which has the highest maximum photosynthetic rate, it is 42.3% higher than that of bare root seedlings, and container-non-fertilized treatment (5.14–6.11 mmol CO_2_·m^−2^·s^−1^) decreased by more than 45.9% (Figure 5d). 

Stomatal conductance (Figure 5e) and stomatal transpiration rate (Figure 5f) also showed similar trends to the maximum photosynthetic rate and increased significantly with higher fertilization concentrations (*p* < 0.001).

Looking at the water utilization efficiency, the higher the fertilization concentration, the higher the significant increase (*p* < 0.001), and showed a relatively low tendency in the bare root seedling and container-non-fertilized treatments (Figure 5g). Overall, the net quantum yield was high at 138.0 g/yr/tree of fertilization treatment and decreased at 207.0 g/yr/tree (Figure 5h) treatment. Bare root seedling cultivation showed the lowest value with 0.018 mmol CO_2_·mol^−1^. In the case of container-non-fertilized treatment, the average was 0.025 mmol CO_2_·mol^−1^, and there was no significant difference from bare root seedling cultivation.

### 2.5. Chlorophyll Fluorescence Change According to Fertilization and Irrigation

**Figure 6:**
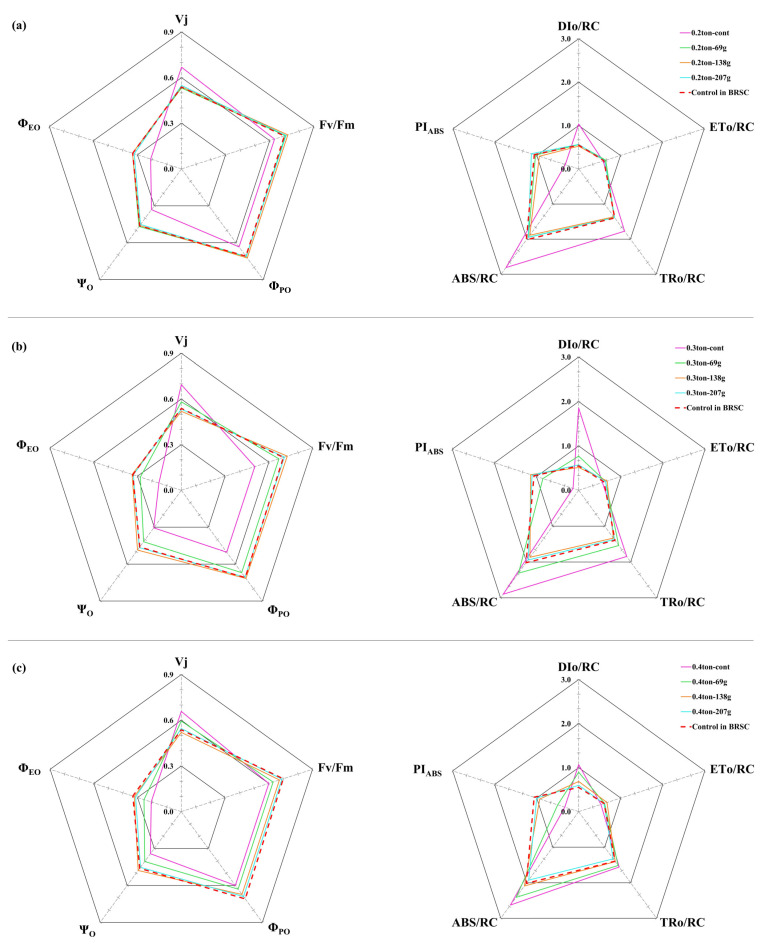
Chlorophyll Fluorescence of containerized seedlings of *H. syriacus* L. grown under three different irrigation and four fertilization treatments from 2020 to 2021. BRSC is an abbreviation for bare root seedling cultivation. (**a**): 0.2 ton/yr/tree irrigation treatment; (**b**): 0.3 ton/yr/tree irrigation treatment; (**c**): 0.4 ton/yr/tree irrigation treatment.

The relative variable fluorescence, Vj showed a high value in container-non-fertilized treatment (0.67 ± 0.01) and increased by 19.8% compared to bare root seedling cultivation (0.54 ± 0.02) (Figure 6). On the other hand, at 0.3 ton-138.0 g/yr/tree (0.52 ± 0.01), a lower value was shown due to a significant (*p* < 0.001) effect of fertilization treatment. It was reduced by 22.3% compared to container-non-fertilized treatment and 3.7% compared to bare root seedling cultivation. Φ_PO_ interacted according to irrigation-fertilization treatment (*p* < 0.001). Φ_PO_ showed the lowest value in container-non-fertilized treatment (0.50~0.63), and as the fertilization concentration increased, Φ_PO_ also increased significantly (*p* < 0.001) in the 0.3 ton-138.0 g/yr/tree (0.72 ± 0.02) treatments showed high values. Ψ_O_ and Φ_EO_ also showed significantly (*p* < 0.001) higher values in the 0.3 ton-138.0 g/yr/tree treatment compared to bare root seedling cultivation and container-non-fertilized treatment.

ABS/RC and DIo/RC increased in bare root seedling cultivation and container-non-fertilized treatment and showed the lowest values at 0.3 ton-138.0 g/yr/tree treatment. On the other hand, TRo/RC and ETo/RC decreased in bare root seedling cultivation and container-non-fertilized treatment. The fertilization concentration increased to 138.0 g/yr/tree and then decreased to 207.0 g/yr/tree. PI_ABS_ was significantly reduced by about 75.5% in container-non-fertilized treatment (0.13~0.33) treatment compared to bare root seedling cultivation (1.06 ± 0.17) (*p* < 0.001). It showed a value 77.2% lower than the highest value of 0.3 ton-138.0 g/yr (1.14 ± 0.08) treatment and showed a significant (*p* < 0.001) difference between containerized seedling cultivation treatments. 

### 2.6. Nitrogen Nutrient Characteristics According to Fertilization and Irrigation 

**Figure 7:**
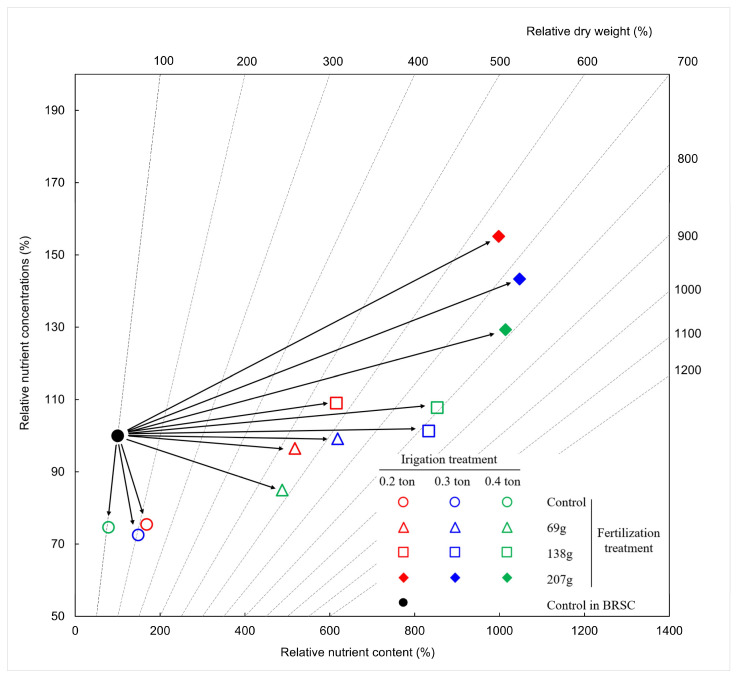
Nitrogen status nutrition distribution from 2020 to 2021 on container seedlings of *H. syriacus* L. grown under three irrigation and four fertilization treatments. BRSC is an abbreviation for bare root seedling cultivation.

Nutrient vector diagnosis results for all treatments showed different trends and fertilization treatment increased growth compared to bare root seedling cultivation and container-non-fertilized treatment (Figure 7). Container-non-fertilized treatment shows a “translocation” state in which element deficiency and growth do not appear, and 0.2, 0.3, 0.4 ton-69.0 g/yr/tree treatment eliminates the growth restriction caused by the deficiency of a specific element by other elements. It showed a state of “dilution of nutrients” that appears when the 0.2, 0.4 ton-138.0 g/yr/tree treatment showed “nutrient deficiency” in which plant nutrient concentration did not increase as much as the plant growth and nutrient content increased, and the 0.3 ton-138.0 g/yr/tree treatment showed growth. While the nutrient content increased, the concentration remained constant, showing a “nutrient optimum” state. In the case of the 0.2, 0.3, 0.4 ton-207.0 g/yr/tree treatment, the concentration increased, but there was no change in growth, resulting in an “excessive nutrient” state in which nutrients were consumed excessively.

## 3. Discussion

### 3.1. Effect of Different Irrigation-Fertilization on Growth Performances

Fertilization treatment significantly improved the height and root collar diameter growth, and 0.3 ton-138.0 g/yr/tree treatment of container seedlings increased 37.8% and 26.5%, respectively, compared to bare root cultivation. Plants can show various growth and biomass production changes through environmental control, such as moisture, light, nutrients, and temperature [25]. In particular, in containerized seedling cultivation, since the nutrient requirements are different for each tree species and season, excessive or insufficient nutrients cause deterioration in the growth and quality of seedlings [26,27]. Fertilization affects soil organic matter content, microbial properties, and enzyme activity, changing growth characteristics [28,29,30]. Fertilization treatment is known to improve the growth of the stem and rhizome [31,32,33], but excessive fertilization can cause a reduction in nutrient utilization efficiency [12], it is necessary to suggest an appropriate level of fertilization depending on the species [20,34,35]. Therefore, further studies should be conducted to confirm whether the cause of the decrease in growth at 207.0 g/yr/tree fertilization was an excess or a symptom of deficiency.

In fertilization treatment, nitrogen is a significant component of amino acids, proteins, and chlorophyll, and the most significant amount of inorganic nutrients is contained in plants. Hence, nitrogen fertilization at an appropriate concentration is significant in containerized seedling cultivation [36]. The nitrogen concentration in the 69.0 g/yr/tree fertilization treatment of Multifeed 20, a water-soluble fertilizer used in this study, was 13.8 g/yr/tree, 138.0 g/yr/tree, and 207.0 g/yr/tree treatment, respectively, and the nitrogen concentration was 13.8 g/yr (34.8 mg/L), 26.7 g/yr (70.0 mg/L), and 41.4 g/yr (104.6 mg/L). The appropriate nitrogen fertilization concentration for each containerized seedling cultivation growth stage in the US Nursery Survey Manual is 12~125 mg/L during the seedling formation period, 55~260 mg/L during the rapid growth period, and 0~141 mg/L during the hardening period [35]. These values are higher than the nitrogen fertilization concentrations in this experiment. On the other hand, Ingested [37] reported that nitrogen concentrations of 50 mg/L during seedling growth, 100 mg/L during rapid growth, and 25 mg/L during hardening were appropriate. This was similar to the nitrogen fertilization concentration of this experiment.

The health of nursery seedlings can be judged by the H/D ratio, which is the ratio of above-ground growth to underground growth [38]. It is known that the higher the H/D ratio, the more vulnerable to stress such as wind, dryness, and low temperature after planting [39,40]. Johnson et al. [41] reported that if the H/D ratio value is less than 10 cm·mm^−1^, containerized seedling cultivation can be classified as a healthy tree. As a result of this study, the H/D ratio values in all treatment groups were less than 10 cm·mm^−1^, which was judged as a healthy seedling. The H/D ratio showed a significant effect on the fertilization treatment, and in particular, the lowest value was shown in the 0.3 ton-138.0 g/yr treatment.

### 3.2. Changes in Biomass Production and Quality of Seedlings Due to Fertilization and Irrigation

Containerized seedling cultivation increased the amount of biomass by 44.5% compared to bare root cultivation. Even in the same container seedling cultivation, biomass production increased as the fertilization amount increased. Proper fertilization in container seedlings can produce seedling of excellent quality and is known to increase the amount of biomass [42]. In addition, proper fertilization according to the nutrient requirements of each species brings optimal nutrient conditions in the soil, and as a result, growth is increased with active photosynthetic activity [43,44,45].

In the case of the T/R ratio, significantly higher values were shown in bare root seedling cultivation and container-non-fertilized treatment. The 0.3 ton-138.0 g/yr/tree treatment showed the lowest value, which had the highest growth and biomass production. It is estimated that the 0.3 ton-138.0 g/yr/tree treatment distributed more photosynthetic products to underground growth than to above-ground growth compared to other treatments. The distribution of photosynthetic products and the development of root systems are known to impact afforestation performance significantly [46,47]. Seedlings with high above-ground biomass production and T/R ratio may show poor planting performance due to poor resistance to drying stress after planting [48].

Containerized seedlings with excellent photosynthetic ability by proper fertilization show tremendous growth and a high survival rate even after planting [49,50]. This is because proper fertilization increases the water absorption and transport capacity of the roots [26,51,52].

SQI, which represents tree quality, showed the highest value in container seedlings treated with 0.3 ton-138.0 g/yr/tree. The QI of container seedling fertilization treatment was higher than that of bare root seedling cultivation and container-non-fertilized treatment. It is believed that this is because the process of distributing photosynthetic products was adequately performed, and the nutrient content and nutrient utilization efficiency in the container were high.

### 3.3. Changes in Flowering Characteristics According to Fertilization and Irrigation

Irrigation-fertilization shortened the flowering period of *H. syriacus* L. and significantly increased the flowering. In particular, when treated with 0.2 ton-207.0 g/yr/tree and 0.3 ton-138.0 g/yr/tree, the flowering period was shortened by 19 days compared to root seedling cultivation, and the flowering rate increased by more than 78.0%. This result is because the continuous fertilization treatment, including nitrogen, increased the flowering rate. Brunkhorst [53] and Berding and Skinner [54] also reported that appropriate fertilization treatment increases the flowering rate, but excessive nitrogen fertilization can delay and decrease the flowering rate.

As for the flowering period, due to the interaction between irrigation-fertilization treatment, flowering was continuously achieved in the treatments of 0.3 ton-207.0 g/yr/tree (91.8 ± 0.3 days) and 0.3 ton-138.0 g/yr/tree (88.3 ± 1.3 days). These results are presumed to be due to various components such as calcium, phosphorus, and nitrogen of Multifeed, a water-soluble fertilizer used in this study. Gosnell [55] reported that the amount of flowering decreased as a result of fertilization with high phosphorus content, whereas Brunkhorst [56], Van Dillewijn [57], and Hale et al. [58] reported that the amount of flowering was greatly improved. Therefore, it is judged that studies should also be conducted to establish an appropriate level of fertilization mix and concentration range that affect future flowering.

### 3.4. Photophysiological Changes According to Fertilization and Irrigation

The photosynthetic capacity of *H. syriacus* L. was reduced in bare root seedling cultivation and container-non-fertilized treatment (Figure 4). The light compensation point increased up to the fertilization concentration of 138.0 g/yr/tree but significantly decreased from 207.0 g/yr/tree. On the other hand, the light saturation point tended to increase as the fertilization concentration increased and decreased in the container-fertilized untreated area. Plant photosynthetic capacity and nitrogen content have a very close relationship [59]. This is because more than 70% of all nitrogen in leaves constitutes photosynthesis proteins [60]. In other words, excess or deficiency of nutrients due to fertilization may cause inhibition of photosynthetic ability in the containerized seedling cultivation process and, in particular, may significantly affect chloroplast production most closely related to photosynthetic activity. According to Yang et al. [61], it has been reported that the light compensation point and light saturation point are lowered when substantial assimilative product accumulation does not occur. In the case of the container-non-fertilized treatment group, it is judged to be the result of the effect of nutrient deficiency.

Dark respiration rate is an important mechanism for supplying carbon, reducing power, and ATP required for biosynthetic processes [62] and tends to decrease when plants are subjected to water stress [63]. The dark respiration rate decreased similarly to the light compensation point. In the case of bare root cultivation, 0.09 ± 0.03 mmol·m^−2^·s^−1^ was reduced by 94.9~95.1% compared to 0.2 ton-138.0 g/yr/tree and 0.3 ton-138.0 g/yr/tree treatments. This result seems to be because bare root cultivation relies on natural rainfall compared to container cultivation, where irrigation is continuously supplied. In other words, it is presumed to have occurred due to unavoidable drying damage despite well-managed bare root seedling cultivation.

The maximum photosynthetic rate increased as the fertilization concentration increased. The maximum photosynthetic rate was significantly higher (*p* < 0.001) in the 138.0 g/yr/tree, and 207.0 g/yr/tree treatments, but the container-control treatment showed a relatively lower maximum photosynthetic rate than the fertilized treatment. In addition, the container-non-fertilized treatment showed a 6.3% lower maximum photosynthetic rate than the bare root cultivation, indicating that the activity of the photochemical system was significantly reduced. Even in the case of oak trees, it was found that the maximum photosynthetic rate was superior to the container-non-fertilized treatment under appropriate fertilization conditions [12]. On the other hand, since the photosynthetic rate is highly related to stomatal opening and closing, stomatal conductance and photosynthetic rate are positively correlated [64,65]. Therefore, low maximum photosynthetic rate and stomatal conductance were observed in bare root seedling cultivation and container-non-fertilized l treatment.

Stomatal conductance, which means the rate of diffusion of water into the atmosphere, is affected by various environmental factors. As stomatal conductance decreases, the rate of stomatal transpiration decreases, and photosynthetic capacity is affected. In particular, the photosynthesis of higher plants is greatly limited by adequate water. When the water supply is limited, plants are stressed, and the ABA (abscisic acid) content increases. Afterwards, stomata begin to close according to the change in ABA content in the plant, which affects CO_2_ absorption and negatively affects photosynthesis [66,67]. The stomatal conductance and transpiration rate were similar to the maximum photosynthetic rate. Stomatal conductance and stomatal transpiration rate decreased by 2.1% and stomatal transpiration rate by 30.0% in the container-non-fertilized treatment compared to bare root cultivation. In the case of the container-non-fertilized treatment, the maximum photosynthetic rate was reduced due to stomatal closure, which also affected the gas exchange process that diffuses CO_2_ in the atmosphere into mesophyll cells.

Water utilization efficiency is closely related to plant growth. It was confirmed that nitrogen fertilization increases the plant growth hormone ABA, a stomatal conductance-regulating hormone, to increase water utilization efficiency by controlling stomata [68]. In this study, the water utilization efficiency increased as the fertilization concentration was higher than that of bare root seedling cultivation and container-non-fertilized l treatment. In particular, the container-non-fertilized treatment group showed the lowest value along with the photosynthetic rate. This is because the supply of CO_2_ and water is greatly restricted due to stomatal closure, and the diffusion resistance of CO_2_ in the cell is also increased, so it is judged that the water utilization efficiency is reduced along with photosynthesis [69,70]. 

In the case of net quantum yield [71], which reflects the activity of the photochemical system, the highest value was shown at 138.0 g/yr/tree of fertilization treatment, which is judged to be higher in the fertilized group than in the non-fertilized group due to the appropriate fertilization conditions. In addition, it can be seen that the activity of the photochemical system was significantly reduced in the bare root cultivation and container-non-fertilized treatment, and in the 207.0 g/yr/tree treatment with high fertilization concentration, it gradually decreased compared to 138.0 g/yr/tree. Therefore, it is judged that the results of the photosynthetic reaction according to irrigation-fertilization treatment for *H. syriacus* L. can be usefully used to set the appropriate fertilization level for each cultivation method.

### 3.5. Chlorophyll Fluorescence Change According to Fertilization and Irrigation

Through the chlorophyll fluorescence reaction, it was found that fertilization treatment in containerized seedling cultivation affects the photoreceptivity in the light reaction process [12]. The higher the relative variable fluorescence, Vj, the lower the reoxidation rate of Q_A_^−^, which means that electron transfer is inhibited, and it is closely related to the inactivation of the oxygen-evolving complex (OEC) [72,73]. Vj showed the lowest value at 0.3 ton-138.0 g/yr/tree, whereas the lowest value was shown at container-non-fertilized treatment, indicating that electron transfer was inhibited compared to bare root cultivation. Φ_PO_, Ψ_O_, and Φ_EO_ mean the energy transfer rate and fluorescence yield for each step of the photochemical reaction [74]. In the case of Φ_PO_, which represents the maximum quantum yield in the initial photochemical reaction, [75], the higher the abiotic stress, the lower it tended to be. In this study, the container-non-fertilized treatment showed the lowest value, and 0.3 ton-138.0 g/yr/tree showed the highest value. Ψ_O_ and Φ_EO_, which are electron transfer efficiencies after Q_A_^−^, also showed low values in container-non-fertilized treatment. Similar to the results of this study, these fluorescence variables were reported to have decreased in stressed plants [76]. In other words, the non-fertilization treatment in container cultivation indirectly shows that the number of reaction centers in an inactive state increases. The most captured energy is discarded rather than sent to the electron transport system.

ABS/RC, TRo/RC and DIo/RC, representing changes in energy flow per reaction center, increased in container-non-fertilized treatment and showed the lowest values at 0.3 ton-138.0 g/yr/tree. The increase in ABS/RC indicates that the reaction center is in a partially inactive state as the number of reaction centers in a reduced state increases [73], and the reaction center in an overall active state through an increase in TRo/RC and DIo/RC It can be seen that as these decreases, both the energy captured per reaction center and the energy released as heat increase. In particular, the increase in DIo/RC is closely related to photoinhibition and can be understood to reduce damage caused by the inflow of excessive excitation energy in stress [77]. ABS/RC and TRo/RC gradually increased compared to the fertilized treatment in container-non-fertilized treatment, indicating that both the amount of light absorbed per leaf area and the energy captured in photosystem II decreased. Among the energies captured in photosystem II, ETo/RC also showed the lowest value in container-non-fertilized treatment, indicating that the energy transmitted through electron transfer is decreasing. On the other hand, in container-non-fertilized treatment, it is accompanied by an increase in DIo/RC and a decrease in Ψ_O_, confirming that photoinhibition is occurring. It can be seen that much energy is wasted without being used by electron transfer.

PI_ABS_ refers to the energy conservation efficiency in reducing electron carriers using absorbed light energy [78]. Three main steps regulate photosynthetic activity in the reaction center of photosystem II., that is, the total density of active reaction centers, the rate at which the photochemical process captures energy absorbed by reaction centers, and electron movement within the electron transport process after Q_A_^−^ reduction are all reflected. It is also known as a more sensitive environmental stress index than the maximum quantum yield (Φ_PO_ = Fv/Fm) of photosystem II [72,74,79,80]. In particular, PI_ABS_ is used as a good indicator for evaluating and monitoring abiotic stress [81], and it is known that PI_ABS_ decreases according to abiotic stress [72,82]. In this study, it was the lowest in container-non-fertilized treatment and high in 0.3 ton-138.0 g/yr/tree. As the energy increased, the activity of photosystem II appeared to decrease.

### 3.6. Nitrogen Nutrient Changes According to Fertilization and Irrigation

Nutrient vector analysis can simultaneously analyze seedlings growth, nitrogen concentration changes, and nitrogen content absorbed by seedlings. It can trace the relationship between growth and nutrient characteristics according to fertilization effects [83]. In this study, each treatment area’s tree quality index and nutrient storage capacity differed due to the difference in irrigation-fertilization effects. It was possible to suggest an increase or decrease in fertilization amount by judging the nutrient condition. In the 0.3 ton-138.0 g/yr/tree treatment, “nutrition optimization” was observed, and growth was 88.2% higher than that of bare root seedling cultivation and 75.3–87.4% higher than that of the entire container-non-fertilized treatment. Salifu and Jacobs [84] also reported that, through nitrogen-based nutrient vector analysis for bare root seedling cultivation of *Quercus rubra*, the non-fertilized control group was in a “nutrient deficient” state but was changed to a “nutrient optimal” state by fertilization treatment. This study’s “nutrient deficiency” state was 0.2, 0.4 ton-138.0 g/yr/tree, and the same fertilization concentration showed differences in nutrient storage capacity according to irrigation treatment. Studies correcting the increase and decrease will need to be done later. “Nutrient excess” appeared at 207.0 g/yr/tree of fertilization treatment, which is judged to have affected the growth of trees because the nutrients accumulated in the 40 L container could not be used for growth activities. Teng and Timmer [85] reported that seedling growth decreased due to increased soil acidity and aluminum toxicity after treatment of white spruce (*Picea glauca*) seedlings with excessive ammonium nitrate. DeVisser and Keltjens [86] reported that Douglas-fir (*Pesudotsuga menziesii*) seedlings treated with ammonium fertilizer reduced root dry weight, which also affected the reduction of above-ground growth. As a result of nutrient vector analysis shows that when *H. syriacus* L. (2-1) is cultivated in a 40 L container, trees with optimal nutrient storage capacity can be produced when treated with 0.3 ton-138.0 g/yr/tree. Salifu et al. [87] reported that the nutrient concentration positively correlated with the growth after planting in a study conducted on open seedlings of *Quercus rubra* and *Q. alba*. In the process of planting, improvement of seedlings’ nutrient storage capacity or increase in nutrient content has little effect on growth, but it has a gradual effect on growth and establishment after planting, improving planting performance [88,89]. In particular, the ability to store nutrients can be demonstrated more in planting sites with poor location environments, such as dry and poor regions [90].

Overall, fertilization significantly improved the growth of *H. syriacus* L. container seedlings, and nitrogen fertilizers at appropriate concentrations were particularly important in container seedling cultivation. In addition, containerized seedling cultivation significantly increased biomass by 44.5% compared to bare root cultivation, suggesting that proper fertilization of containerized seedlings is important for good seedling quality and biomass production. Appropriate fertilization and irrigation (0.2 ton-207.0 g/yr/tree and 0.3 ton-138.0 g/yr/tree treatments) positively affected the flowering period and rate, as well as maximum photosynthetic rate and photoreceptivity. Due to the irrigation-fertilization effect, the tree quality index and nutrient storage capacity were also improved. This result suggests that fertilization and irrigation should be considered in producing container seedlings.

## 4. Materials and Methods

### 4.1. Experimental Site Description

**Figure 8:**
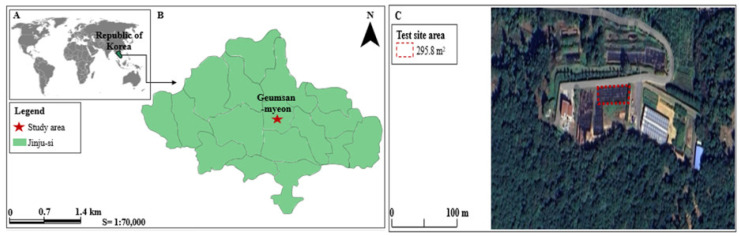
Location of the study site in Jinju-si (**A**) Location of Republic of Korea (**B**) Study site location within the Jinju-si (**C**) Experimental site.

The experiment was conducted from 3 April 2020 to 30 October 2021 in the test forest (Latitude 35°21′ N, Longitude 128°15′ E, Altitude 521 m above sea level) at the Forest Biomaterials Research Center of the National Institute of Forest Science, Jinju-si, Gyeongsangnam-do, Korea (Figure 8). The average annual temperature in Jinju is 15.9 ± 9.6 °C, the highest temperature in the past 10 years is 37.1 °C, and the lowest temperature is −15.3 °C. The climate of the research site is clear and dry in spring and autumn due to the influence of the migratory anticyclone. Summer is hot and humid due to the influence of the north pacific anticyclone, and cold and dry in the winter due to the influence of the cold weather [91]. The annual precipitation was 2800 mm, and during the experimental period (517 days), the period without rain was 362 days, and the days with rain were 155 days.

### 4.2. Irrigation-Fertilization

**Figure 9:**
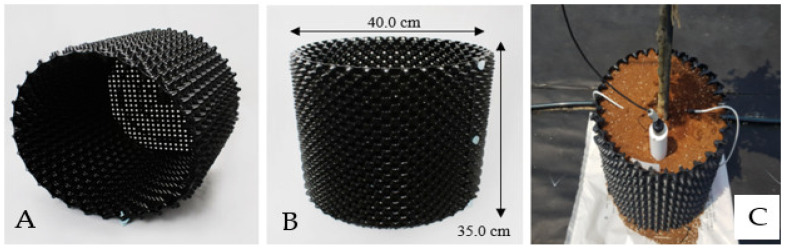
Container used for the experiment. (**A**,**B**): 40L (D40x0H350) porous plastic container, (**C**): Semi-automatic drip irrigation using inverter pump and electric control valve.

**Figure 10:**
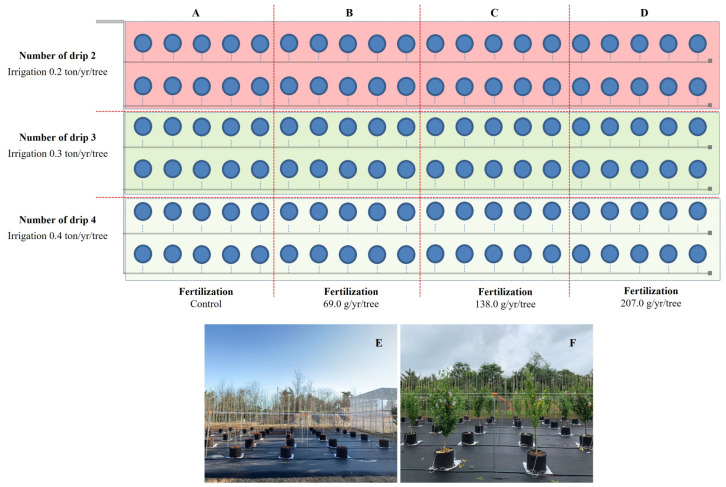
Fertilization and irrigation level pot experimental design. All treatment were repeated 10 times. (**A**): Fertilization control (0.2, 0.3, 0.4 ton), (**B**): 69.0 g (0.2, 0.3, 0.4 ton), (**C**): 138.0 g (0.2, 0.3, 0.4 ton), (**D**): 207.0 g (0.2, 0.3, 0.4 ton), (**E**): *H. syriacus* L. growing in the test site (March) and (**F**): *H. syriacus* L. growing in June.

The test material was a 3-year-old hardwood cutting propagation of *Hibiscus syriacus* L. for. *Haeoreum*, a fast-growing cultivar for street trees (Figure 9). Samples were supplied by the Forest Bioresources Department of the National Institute of Forest Science (NIF_O_S) in March 2020 and used in the study. Individuals with excellent traits were selected, and after removing the soil on the roots with high-pressure water, they were transplanted into a 40L (D400xH350) porous plastic container (Rootpluse, Jeongeup, Korea) (Figure 10). The soil used for transplantation was peat moss:perlite: vermiculite = 1:1:1 (*v*/*v*) mixed soil (Table 3), and survival was confirmed for one month after transplantation, and individuals in good growth conditions (height 1.4 ± 0.1 m, underground 0.4 ± 0.0 m, root diameter 24.4 ± 5.6 cm) were selected and used in the experiment, 10 per treatment group (90 in total).

Fertilization was carried out twice a week from 3 April 2020 to 30 August 2021 (fertilization was not treated from September 2020 to March 2021). A water-soluble fertilizer, Multifeed 20 (N:P:K, 20:20:20, Haifa Chemical Co., Haifa, Israel), was used. Fertilization treatment was carried out at five treatments, including control 2 treatments (no treatment for bare root seedling cultivation and container-non-fertilized cultivation) and treatment 3 (69.0, 138.0, 207.0 g/year/tree) (Table 4). The fertilization treatment consisted of 5 treatments, including 2 control treatments and 3 fertilization treatments. Controls were marked as untreated (BRSC) and container-non-fertilized treatment in bare root seedling cultivation (Figure 10).

Irrigation was carried out by a semi-automatic drip irrigation method using an inverter pump (PBI-(L)803MA/P, Wilo, Busan, Korea) and an electric control valve (IR 200, Bermed, Israel). A dropper spraying 2 L of water per hour was installed at a depth of 5 cm from the soil’s surface in the container, and the amount of irrigation was controlled by the number of droppers installed and the irrigation time. The amount of irrigation was divided into three levels: 1.5, 2.2, and 2.9 ton/year, and the irrigation cycle was treated as 2 times/week from April to May, 7 times/week from June to August, and 2 times/week from September to October. (Table 5). Irrigation-fertilization occurred between 8 and 10 am to reduce damage from high temperatures and sunlight (Figure 10). 

### 4.3. Growth Performances Investigation

Growth surveys for fertilization and irrigation treatment were conducted at 4-month intervals from early March 2020 to mid-September 2021. Growth and relative growth were analyzed by repeatedly measuring root collar diameter and height for 90 seedlings, 10 for each treatment. The relative growth rate for each unit period (day^−1^) and total growth period for the measured root collar diameter and height values before treatment was calculated [92].

In addition, the H/D ratio [SQ; sturdiness quotient = height (cm)/root collar diameter (mm)] was calculated [93]. Root collar diameter and height were measured using steel tape and electronic calipers. After all, experiments were completed in mid-September 2021, 5 seedlings were collected for each treatment, divided into above ground and underground parts, dried at 65 °C for 72 h in a dryer, and then each dry weight was measured. Based on the dry weight measurement results, each biomass, total material production and distribution ratio, and T/R ratio were calculated [94]. In addition, the Seeding Quality Index (SQI: Seeding dry weight (g)/(H/D ratio + T/R ratio) representing the quality index for each grown object was calculated [38].

### 4.4. Flowering Characteristics Investigation

The standard of the flowering period was calculated as the number of days until the end of flowering when more than 70% of the flowers fell from a tree based on the time when the flowering width was more than 50%. The flowering amount (ea/day) was measured by the total number of flowers from the beginning of flowering to the end of flowering. In addition, the mean flowering time (MFT) and flowering speed (FS) were investigated according to the flowering period, and the calculation formula is as follows. Here, t_i_ is the number of irradiation days after flowering, n_i_ is the number of flowers on the day of irradiation, and N is the total number of flowers that have bloomed.

MFT(ea/day) = Σ(t_i_n_i_)/N (1)

FS(ea/day) = Σ(t_i_n_i_) (2)

### 4.5. Photosynthetic Characteristics Investigation

The photosynthetic investigation according to each treatment was conducted on healthy leaves of individuals with similar growth rates and average growth conditions for each treatment from 2020 to 2021. Photosynthesis was measured in August 2021 using a portable photosynthesis system (Portable photosynthesis system, Li-6400, LI-COR Inc., Lincoln, NE, USA). The photosynthetic reaction was measured from 9:00 am to 2:00 pm with a different PPFD of 9 levels. PPFD (Photosynthetic Photon Flux Density) was set to 0, 50, 100, 300, 500, 800, 1000, 1500 and 2000 μmol·m^−2^·s^−1^ using an LED light source (LI-6400-02, LI-COR Inc., Lincoln, NE, USA). The flow rate of the air flowing into the leaf chamber of the photosynthesis meter was set at 400 μmol·m^−2^·s^−1^, and the temperature was set at 25 °C to prevent the influence of environmental changes in the outside air. In addition, by attaching the CO_2_ injector system (LI-6400-01, LI-COR Inc., USA) to the photosynthesis meter, the CO_2_ concentration was measured at 400 μmol^−1^. And a stable state was maintained within the range of ±2 μmol·mol^−1^ [59,65,95]. By measuring the photosynthetic rate by light intensity, the net photosynthetic rate per unit leaf area (Pn), stomatal transpiration rate (E), light compensation point, light saturation point, the apparent quantum yield, etc. were measured 5 times per individual in each treatment, and the average value was obtained.

All measured data were automatically stored in the Data Logger. The net photosynthetic rate, stomatal transpiration rate, light compensation point, and light saturation point per unit leaf surface area were calculated and displayed using the formula of Caemmerer and Farquhar [96] and the light-photosynthesis curve. It was expressed by the following Kume and Ino [97] equation.

Pn = Pn max [1 − exp(−ΦP/Pm max)] − R(3)

Here, Pn = net photosynthetic rate, Φ = net quantum yield, P = luminous intensity, Pn max = maximum photosynthetic rate, and R = arc rate.

The apparent quantum yield, an indicator of photosynthetic ability under weak light conditions, was obtained by the linear regression equation y = ax + b in the light intensity range of 0 to 100 μmol·m^−2^·s^−1^. Here, a is the net quantum yield, the y-intercept b is the dark respiration rate, the x-intercept -b/a is the light compensation point, and the light saturation point is the light intensity at which the relative photosynthetic rate is 80% or more. 

### 4.6. Chlorophyll Fluorescence Investigation

Chlorophyll fluorescence reaction analysis was performed in OJIP (polyphasic rise of chlorophyll fluorescence transients) in August 2021. The average value was presented by measuring 5 times per individual in each treatment group at the same time as the photosynthesis investigation. The measurement was performed using a chlorophyll fluorometer (OS-5P, Opti-Sciences Inc., Hudson, NH, USA) and 3500 μmol·m^−2^·s^−1^ light intensity was applied to the dark-adapted leaves for 20 min. It was irradiated for 1 s, and the chlorophyll fluorescence density was irradiated at 50 μs (O stage), 2 ms (J stage), 30 ms (I stage), and 300 ms (P stage) [98]. OJIP analysis showed that biophysical parameters (Φ_PO_, Φ_EO_, Ψ_O_, ABS/RC, DIo/RC, TRo/RC, ETo/RC, PI_ABS_) were calculated and presented [74] (Table 6).

### 4.7. Investigation of Nitrogen Nutrient Characteristics

**Figure 11:**
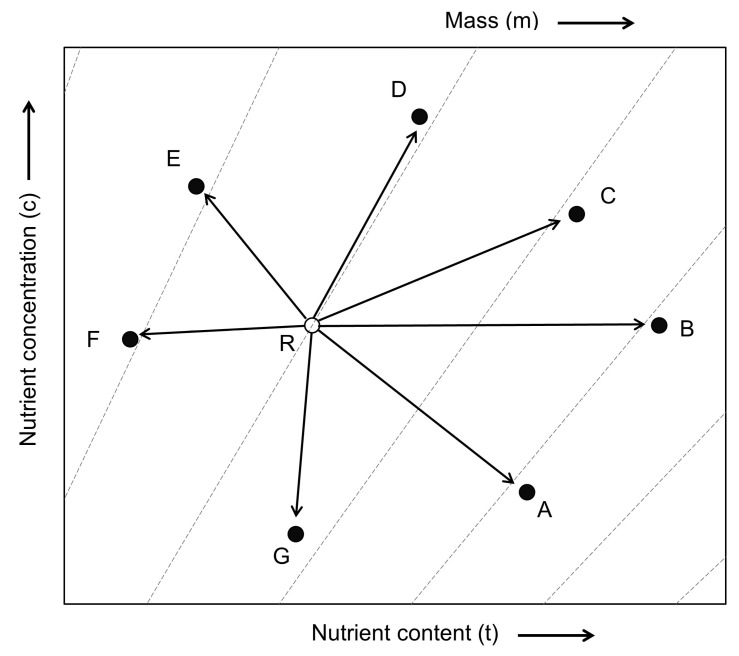
Vector interpretation of directional changes in relative dry mass (m), nutrient content (t), and nutrient concentration (c) of plants grown at different fertilizer applications. Vector length reflects the magnitude of differences among individual plant parameters (modified from [83,103]). A-G and R are described in Table 7.

To analyze nitrogen nutrient characteristics according to irrigation-fertilization treatment in container cultivation, 5 seedlings for each treatment were selected after September 2021, and divided into roots, stems, and leaves. Each dry weight and nutrient concentration were investigated. For the nitrogen nutrient concentration of the individual tree, the plants were divided into parts and dried at 90 °C for more than 72 h. The dried sample was finely ground and measured after burning at a high temperature of 1200 °C using a CN analyzer (Variomax CN, ELEMENTAR, Langenselbold, Germany) [99,100]. In addition, vector analysis [101,102] was performed using the plants’ nutrient concentration, content, and dry weight investigated to investigate the relationship between nitrogen and nutrient changes. In the vector analysis, the length of the arrow indicates the size of the plant’s response according to the nutrient conditions for each treatment (Figure 11, Table 7). “Nutrient deficiency” was judged when plant nutrient concentration did not increase as much as plant growth and nutrient content increased (vector C), and “nutrient optimum” was determined when the concentration remained constant during growth and nutrient content increased. (Vector B). If the nutrient concentration increases but there is no change in growth, it is judged as a “nutrient excess” state in which nutrients are consumed excessively (vector D), and if the growth decreases, it is “excess accumulation” or “nutrient toxicity” by nutrients. (vector E or F). “Nutrient dilution” (vector A) was judged to occur when growth restrictions due to deficiency of a particular element are resolved with another element. Roots, stems, and leaves of plants used to measure nutrient concentrations were samples collected after September 2021.

### 4.8. Statistical Analysis

Statistical analysis of experimental results was tested using Duncan’s Multiple Range Test (DMRT, *p* < 0.05) at a significance level of 5% using R (v 4.0.4) and Sigmaplot 10.0 (SYSTAT, San Jose, CA, USA). All statistical analyzes were performed with SPSS software (ver. 27.0; SPSS Inc., Chicago, IL, USA) was used.

## 5. Conclusions

This study investigated changes in growth and physiological responses and nitrogen nutrient characteristics of *H. syriacus* L. for. *Haeoreum*, a major ornamental tree in Korea, according to irrigation-fertilization. The irrigation and fertilization complex greatly influenced growth-related factors. Most growth-related factors, such as growth and biomass production, were higher in the 0.3 ton-138.0 g/yr irrigation-fertilization treatment. The higher the fertilizer concentration, the higher the flowering rate and the longer the flowering period. In addition, the irrigation and fertilization complex factors were greatly influenced by photosynthetic capacity and chlorophyll fluorescence response. In the case of photosynthetic characteristics, the total photosynthetic capacity was higher in the container fertilization treatment than in the non-water seedling and container-free treatment. In addition, in the case of bare root seedling cultivation and container fertilization treatment, the photosynthetic rate and water utilization efficiency were lowered, and the phenomenon appeared under plant stress. Overall, however, container seedling cultivation was superior to bare root cultivation in terms of growth, photosynthetic capacity, photochemical efficiency, and growth under appropriate nutrient conditions. Our results indicate adequate fertilizer and irrigation are essential for good seedling production. The results of this study will contribute to the standardization of industrial production of superior hibiscus seedlings by scientifically investigating fertilization and irrigation that depend on experience. It is also expected to contribute to producing excellent container seedlings of other tree plants besides *H. syriacus* L.

## Figures and Tables

**Table 1:** Plant height, root collar diameter and H/D ratio measured from 2020 to 2021 on containerized seedlings of *H. syriacus* L. grown under three different irrigation and four fertilization treatments.

Irrigation (ton/yr/tree)	Fertilization	Height		Root Collar Diameter		H/D Ratio (cm·mm^−1^)
		Growth (cm)	Relative Growth Rate (cm·day^−1^)	Growth (mm)	Relative Growth Rate (mm·day^−1^)	
Control in BRSC ^z^		165.60 ± 21.02 ^c^	0.0767 ± 0.0207 ^b^	30.58 ± 6.61 ^d^	0.0025 ± 0.0003 ^de^	5.53 ± 0.69 ^abc^
0.2	Control	195.80 ± 15.26 ^ab^	0.0895 ± 0.0276 ^ab^	34.82 ± 5.96 ^cd^	0.0026 ± 0.0002 ^cde^	5.79 ± 1.28 ^ab^
	69 g/yr	207.33 ± 9.00 ^ab^	0.1062 ± 0.0256 ^ab^	37.81 ± 4.77 ^a-d^	0.0030 ± 0.0002 ^abc^	5.58 ± 0.91 ^abc^
	138 g/yr	196.33 ± 9.71 ^ab^	0.1230 ± 0.0199 ^a^	46.47 ± 1.89 ^ab^	0.0032 ± 0.0001 ^ab^	4.23 ± 0.38 ^c^
	207 g/yr	221.67 ± 6.35 ^a^	0.1220 ± 0.0056 ^a^	46.65 ± 4.30 ^ab^	0.0031 ± 0.0000 ^ab^	4.77 ± 0.36 ^abc^
0.3	Control	192.00 ± 6.25 ^b^	0.1142 ± 0.0262 ^ab^	32.84 ± 3.86 ^cd^	0.0024 ± 0.0004 ^e^	5.92 ± 0.88 ^a^
	69 g/yr	222.67 ± 24.19 ^a^	0.1196 ± 0.0322 ^a^	37.39 ± 4.93 ^bcd^	0.0032 ± 0.0001 ^ab^	6.00 ± 0.69 ^a^
	138 g/yr	208.00 ± 11.36 ^ab^	0.1233 ± 0.0253 ^a^	42.31 ± 8.24 ^abc^	0.0034 ± 0.0003 ^a^	4.02 ± 0.80 ^abc^
	207 g/yr	212.00 ± 6.25 ^ab^	0.1136 ± 0.0303 ^ab^	46.84 ± 7.77 ^ab^	0.0032 ± 0.0002 ^ab^	4.61 ± 0.80 ^abc^
0.4	Control	161.67 ± 22.50 ^c^	0.0988 ± 0.0112 ^ab^	28.52 ± 8.24 ^d^	0.0029 ± 0.0003 ^bcd^	5.82 ± 0.86 ^ab^
	69 g/yr	207.67 ± 27.21 ^ab^	0.1069 ± 0.0264 ^ab^	42.52 ± 3.90 ^abc^	0.0028 ± 0.0002 ^b-e^	4.90 ± 0.63 ^abc^
	138 g/yr	216.75 ± 17.25 ^ab^	0.1094 ± 0.0162 ^ab^	45.44 ± 3.99 ^ab^	0.0030 ± 0.0003 ^abc^	4.43 ± 0.64 ^abc^
	207 g/yr	208.50 ± 5.51 ^ab^	0.1013 ± 0.0116 ^ab^	47.81 ± 6.37 ^a^	0.0029 ± 0.0000 ^a^	4.81 ± 0.67 ^bc^
Irrigation (I)		1.58 ^NS^	1.10 ^NS^	0.29 ^NS^	0.03 ^NS^	0.78 ^NS^
Fertilization (F)		9.90 ***	1.03 ^NS^	14.49 ***	15.66 ***	5.50 **
I × F		2.60 *	0.49 ^NS^	0.76 ^NS^	2.68 *	0.52 ^NS^

^z^ BRSC is an abbreviation for bare root seedling cultivation. ^NS^ *p* ≥ 0.05, * *p* < 0.05, ** *p* < 0.01, *** *p* < 0.001. The H/D ratio was calculated as height (cm)/root collar diameter (mm), and bare root seedling cultivation was indicated as BRSC. Values with different letters in a column indicate statistical differences three irrigation and four fertilization treatments at the 5% levels by Duncan’s multiple range test.

**Table 2:** Flowering of measured from 2020 to 2021 on containerized seedlings of *H. syriacus* L. grown under different three irrigation and four fertilization treatments.

Irrigation	Fertilization	Flowering Beginning (Day)	Flowering Speed	Amount of Flowering (ea)	Flowering Periods
Control in BRSC ^z^		97.8 ± 0.7 ^b^	0.9 ± 0.3 ^fg^	113.0 ± 33.8 ^ef^	68.2 ± 4.5 ^d^
0.2 ton/yr/tree	Control	102.5 ± 1.1 ^b^	0.3 ± 0.1 ^g^	32.0 ± 7.0 ^f^	59.6 ± 2.3 ^e^
	69 g/yr	88.9 ± 9.7 ^c^	1.7 ± 0.8 ^ef^	194.3 ± 82.7 ^de^	78.6 ± 10.9 ^c^
	138 g/yr	82.2 ± 5.0 ^cd^	3.1 ± 0.5 ^bcd^	345.7 ± 56.6 ^bc^	85.3 ± 4.5 ^abc^
	207 g/yr	78.4 ± 0.3 ^d^	3.4 ± 0.2 ^a-d^	362.0 ± 35.8 ^abc^	88.3 ± 2.6 ^ab^
0.3 ton/yr/tree	Control	98.5 ± 0.7 ^b^	0.2 ± 0.0 ^g^	19.5 ± 5.0 ^f^	62.5 ± 0.7 ^de^
	69 g/yr	86.6 ± 3.1 ^c^	2.7 ± 0.7 ^cd^	325.0 ± 80.0 ^bc^	83.9 ± 3.3 ^abc^
	138 g/yr	78.7 ± 0.2 ^d^	3.8 ± 0.5 ^ab^	443.3 ± 70.7 ^ab^	88.4 ± 1.3 ^ab^
	207 g/yr	82.0 ± 1.7 ^cd^	4.1 ± 0.5 ^a^	468.7 ± 57.5 ^a^	91.8 ± 0.3 ^a^
0.4 ton/yr/tree	Control	123.8 ± 3.2 ^a^	0.1 ± 0.0 ^g^	2.5 ± 0.7 ^f^	22.3 ± 3.2 ^f^
	69 g/yr	88.4 ± 0.2 ^c^	2.4 ± 0.5 ^de^	292.3 ± 67.0 ^cd^	82.0 ± 0.3 ^bc^
	138 g/yr	82.1 ± 5.2 ^cd^	3.1 ± 0.4 ^bcd^	342.0 ± 45.3 ^bc^	87.5 ± 4.8 ^abc^
	207 g/yr	85.9 ± 4.0 ^cd^	3.5 ± 0.9 ^abc^	381.0 ± 102.36 ^abc^	83.6 ± 4.8 ^abc^
Irrigation (I)		13.76 ***	4.13 *	4.87 *	21.76 ***
Fertilization (F)		71.03 ***	69.31 ***	59.07 ***	120.63 ***
I × F		5.63 **	0.82 ^NS^	0.99 ^NS^	12.96 ***

^z^ BRSC is an abbreviation for bare root seedling cultivation. ^NS^ *p* ≥ 0.05, * *p* < 0.05, ** *p* < 0.01, *** *p* < 0.001. Values with different letters in a column indicate statistical differences three irrigation and four fertilization treatments at the 5% levels by Duncan’s multiple range test.

**Table 3:** Physical and chemical properties of media used in container seeding production.

Soil Media Mixture (*v*/*v*)			pH	EC (dS·m^−1^)	NO^−^ (mg/kg^−1^)	P_2_O_5_ (mg/kg^−1^)	Ex-Cations (cmol·kg^−1^)			C.E.C (cmol·kg^−1^)
Peat Moss	Perlite	Vermiculite					K^+^	Ca^2+^	Mg^2+^	
1	1	1	6.0	0.1	0.1	123	4	10	7	17

**Table 4:** Fertilization treatment applied to the experiment from 1 May 2020 to 30 September 2021.

Total Fertilization Amount (g/year/tree)	Monthly Fertilization Amount (g/month/tree)						
	April	May	June	July	August	September	October
69.0	-	7.67	7.67	7.67	7.67	7.67	-
138.0	-	15.33	15.33	15.33	15.33	15.33	-
207.0	-	23.00	23.00	23.00	23.00	23.00	-

**Table 5:** Irrigation treatment applied to the experiment from 1 April 2020 to the 30 October 2021.

Number of Drip	Total Irrigation Amount (ton/year/tree)	Monthly Irrigation Amount (L/month/tree)						
		April	May	June	July	August	September	October
2	0.2	24.0	27.0	42.9	44.3	44.2	27.0	27.0
3	0.3	36.0	40.5	64.4	66.5	66.3	40.5	40.5
4	0.4	48.0	54.0	85.9	88.7	88.4	54.0	54.0

**Table 6:** Summary of chlorophyll fluorescence parameters from OJIP test.

Parameters	Description
Vj	Relative variable fluorescence at time (2 ms)
Φ_PO_	Probability that an absorbed photon leads to reduction further than Q_A_^−^
Φ_EO_	Probability that an absorbed photon leads to electron transport further than Q_A_^−^
Ψ_O_	Probability that an absorbed photon leads to reduction of Q_A_^−^
ABS/RC	Absorption flux per reaction center
TRo/RC	Trapping of electrons per reaction center
ETo/RC	Electron flux per reaction center beyond Q_A_^−^
DIo/RC	Energy dissipation flux per reaction center
PI_ABS_	Performance index on absorption basis.

**Table 7:** The interpretation for vector diagnosis. The reference status (R) is usually normalized to 100. Vector shifts (A to G) indicate an increase (+), decrease (−) or no change (0) in dry mass and nutrient status relative to the reference status [104].

Vector Shift	Change in Relative			Possible Diagnosis	Interpretation
	Mass	Concentration	Content		
A	+	−	+	Non-limiting	Dilution
B	+	0	+	Non-limiting	Sufficiency
C	+	+	+	Limiting	Deficiency
D	0	+	+	Non-toxic	Luxury consumption
E	−	++	±	Toxic	Excess
F	−	−	−	Antagonisric	Excess
G	0, −	−	−	Retranslocation	Depletion

## Data Availability

Not applicable.
